# Influence of zoledronic acid and low-intensity laser on collagen fibers during the bone repair process

**DOI:** 10.1590/acb393724

**Published:** 2024-07-15

**Authors:** Paola Aguilar, Angélica Cristina Fonseca, Gustavo Pompermaier Garlet, Jéssica Lemos Gulinelli, Pâmela Letícia Santos

**Affiliations:** 1 Universidade de Araraquara – Department of Health Sciences – Dental School – Araraquara (SP) – Brazil.; 2 Universidade de São Paulo – Department of Biological Science – São Paulo (SP) – Brazil.; 3 Oral sin – Oral and Maxillofacial Surgery – Londrina (PR) – Brazil.

**Keywords:** Bisphosphonates, Lasers, Rats, Bone and Bones

## Abstract

**Purpose:**

To evaluate collagen fibers during the bone repair process in critical defects created in the tibias of rats, treated with zoledronic acid (AZ) associated with low-level laser therapy (LLLT).

**Methods:**

Ten rats were distributed according to treatment: group 1) saline solution; group 2) LLLT; group 3) AZ; group 4) AZ and LLLT. AZ was administered at the dose of 0.035 mg/kg at fortnightly intervals over eight weeks. Next, 2-mm bone defects were created in the tibias of all animals. The bone defects in groups 2 and 4 were irradiated LLLT in the immediate postoperative period. After periods 14 and 28 of application, the animals were euthanized, and birefringence analysis was performed.

**Results:**

Approximately 90% of the total area was occupied by collagen fibers within the red color spectrum, this area being statistically larger in relation to the area occupied by collagen fibers within the green and yellow spectrum, in the four groups. Over the 14-day period, there was no statistically significant difference between the groups. In the 28-day period, group 2 (14.02 ± 15.9%) was superior in quantifying green birefringent fibers compared to group 1 (3.06 ± 3.24%), with *p* = 0.009.

**Conclusions:**

LLLT associated with ZA is effective in stimulating the neoformation of collagen fibers. The LLLT group without the association with ZA showed a greater amount of immature and less organized matrix over a period of 28 days.

## Introduction

The success of bone tissue regeneration is related to local and systemic factors, as well as the dimensions of the bone defect. Therefore, technologies have been studied for tissue regeneration in injured regions^1^^–^ 3.

Among the systemic factors, zoledronic acid stands out, which is an antiresorptive medication, from the bisphosphonate class. This medication works by binding to hydroxyapatite and inhibiting osteoclast recruitment pathways. Due to its performance, the zoledronic acid causes an increase in the speed of osteoclastic apoptosis, and consequently there is a reduction in bone resorption and maintenance of bone mineral density^4^^–^ 6. The bone repair process using zoledronic acid has already been evaluated through clinical, histological, imaging behavior, immunohistochemistry technique, and mechanical tests^2^^,^ 4 ^,^ 5 ^,^ 7.

In relation to local factors, the application of low-level laser stands out, which stimulates the dynamics of the repair process, as it stimulates the differentiation of mesenchymal cells into osteoblasts, activates osteogenic factors, stimulates bone neoformation, the synthesis of collagen and angiogenesis, in addition to improving mechanical properties and reducing the inflammatory reaction^8^^,^ 9.

In the literature, there is little research evaluating the association of zoledronic acid and laser therapy in the bone repair process^3^, alveolar^10^, and with biomaterials^11^. Additionally, none of the aforementioned studies evaluated the quality and quantity of collagen fibers, which are directly related to the bone repair process. Like this it is justified to carry out this study to evaluate collagen fibers during the bone repair process in critical defects created in the tibias of rats, treated with zoledronic acid (AZ) associated with low-level laser therapy (LLLT), through histochemical analysis.

## Methods

The methodology followed the study previously carried out by the research group^3^. Twenty female rats (*Rattus norvegicus* albinus, Wistar) were used. Throughout the experimental period, the animals remained in the vivarium, under controlled temperature conditions (22 ± 2 °C) and a 12-hour light/dark cycle, receiving water and food without restriction.

The project was submitted and approved by the Animal Research Ethics Committee, with protocol number 006/13.

### Experimental design

**Figure 1:**
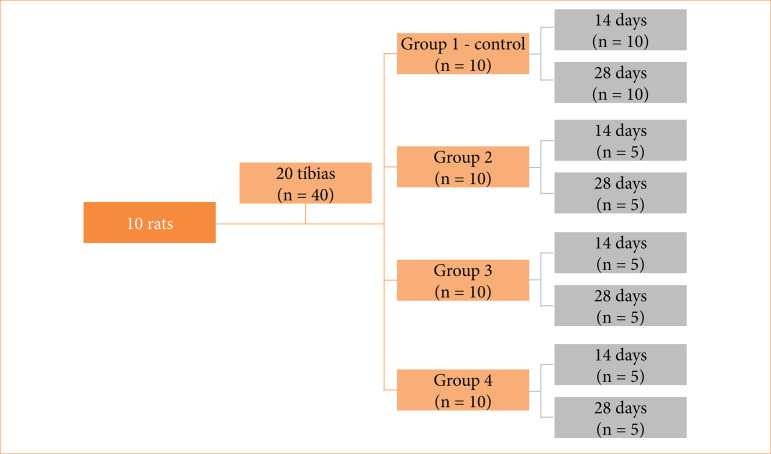
Flowchart for exemplification of groups division.

Source: Elaborated by the authors.

The animals were distributed into four groups, according to the treatment received after the creation of the bone defect in the tibia (n = 10). (Fig. 1).

- Group 1 (control): intravenous administration of saline solution (sodium chloride 0.9%®, Darrow, Rio de Janeiro, RJ, Brazil);
- Group 2: intravenous administration of saline solution and LLLT application at the site of the bone defect (Photon Laser®, DMC, São Carlos, SP, Brazil);
- Group 3: intravenous administration of ZA (Zometa®, Novartis Pharma AG, Basel, Switzerland);
- Group 4: intravenous administration of ZA and LLLT at the site of the bone defect.

The rats in groups 1 and 2 received 0.9% saline intravenously to simulate the stress of ZA application. The animals in Groups 3 and 4 were subjected to intravenous administration of ZA at the dose of 0.035 mg/kg administered intravenously fortnightly, for eight weeks, continuing until the last experimental period.

Fifty-six days after the start of administration of the bisphosphonate, the surgical procedure was performed to create bone defects in the tibias of the animals in all groups studied^3^^,^ 12.

### Surgical procedure

The animals were subjected to sedation through intramuscular injection of the anesthetic 1% ketamine at the dose of 50 mg/kg (Francotar, Virbac LTDA, São Paulo, Brazil) associated with the sedative 2% xylazine hydrochloride at the dose of 5 mg/kg (Virbaxyl 2%, Virbac LTDA, São Paulo, Brazil), the dosage recommended by the manufacturer.

Next, trichotomy of the right and left tibias was performed, and antisepsis of the region to be incised with polyvinyl pyrrolidone iodine degermante (PVPI 10%, Riodeine Degermante, Rioquímica, São José do Rio Preto, SP, Brazil), associated with topical PVPI. After degermation, the animals received local anesthesia using mepivacaine hydrochloride (0.3 mL/kg, scandicaine 2% with adrenaline 1:100,000, Septodont, France) to assist in hemostasis of the operative field.

**Figure 2:**
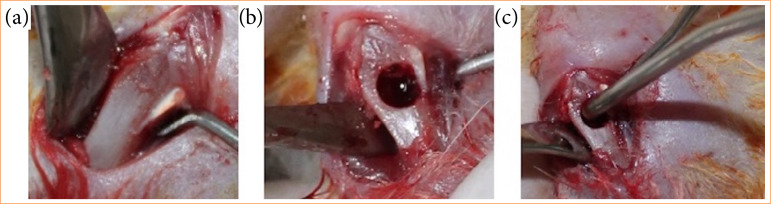
Surgical procedure: 226 × 70 mm (300 × 300 DPI).

Source: Elaborated by the authors.

With a number 15c blade (Feather Industries Ltd, Tokyo, Japan) mounted on a scalpel handle number 3 (Hu-Friedy, Germany), an incision of approximately 1 cm in length was made in the medial portion of the tibias, up to the bone base. Then, the soft tissue was dissected and removed with the help of periosteal detachers, exposing the bone tissue (Fig. 2a).

Subsequently, a bone defect was created with a diameter of 2 mm and a depth of 4 mm in the upper cortex of the rats’ tibias, which is considered a critical defect for the bone dimensions of this animal. Bone defect preparation was performed through ostectomy using a lance drill coupled to an electric motor with constant irrigation with 0.9% sodium chloride (Darrow, Rio de Janeiro, Brazil), throughout the preparation (Fig. 2b).

The soft tissues were carefully repositioned and sutured in planes using absorbable thread (Polygalactina 910–Vycril 4.0, Ethicon, Johnson, São José dos Campos, SP, Brazil) with continuous stitches in the deep plane and with monofilament thread (nylon 5.0, Ethicon, Johnson, São José dos Campos, SP, Brazil) with interrupted stitches in the most external plane, obtaining primary closure of the wound. After suturing, the area was antisepsis again with polyvinyl pyrrolidone, topical iodine (PVPI 10%, Riodeine, Rioquímica, São José do Rio Preto, SP, Brazil).

In the postoperative period, the animals received intramuscular administration of pentabiotic (0.1 mL/kg, Fort Dodge Saúde Animal LTDA, Campinas, SP, Brazil) with a dose in the immediate postoperative period and dipyrone sodium (1 mg/kg/day, Ariston Indústrias Químicas e Farmacêuticas LTDA, São Paulo, Brazil) totaling three doses. No food or movement restrictions were imposed on the animals, which were kept in individual cages throughout the experiment.

In periods of 14 and 28 days after the surgical procedure to create bone defects, the animals were euthanized to remove the specimens.

### Low-level laser therapy

The animals in groups 2 and 4, immediately after milling, were subjected to irradiation with aluminum gallium arsenide laser (ArAlGa), (Photon Laser, DMC, São Carlos, SP, Brazil), with a wavelength of 808 nm, spot size of 0.07 cm^2^, power of 0.03 W for 133 seconds per point, irradiance of 0.42 W/cm^2^ and energy of 4 J/point (57.14 J/cm^2^/point). The area received the total of 32 J of energy. The application was performed once at eight points around the bone defect, in contact with the bone tissue, and also at a central point of the bone defect. In groups 1 and 3, there was no laser application (Fig. 2c).

### Birefringence analysis

The sections were deparaffinized in xylene, hydrated in a decreasing gradient of ethanol and incubated with 0.2% phosphomolylic acid for 10 min to neutralize elastin birefringence. Afterwards, the sections were washed in distilled water and immersed for 90 min in a 0.1% sirius red solution dissolved in saturated aqueous picric acid. Subsequently, the sections were washed in 0.01 HCl for 2 min. Then, the slides were dehydrated in an increasing gradient of ethanol, cleared in xylene and mounted with Entellan^®^.

All fields included in the area of interest were analyzed, used to capture images with a polarizing lens coupled to a binocular inverted microscope (Leica DM IRB/E).

Quantification of the intensity of the birefringence glow was performed using the KS 300/400 AxioVision software (version 4.8, Carl Zeiss). After defining the spectra for green, yellow and red, following RGB values, the images were binarized for each spectrum and the quantity in pixels^2^ of each color in the delimited total area of interest was measured^13^.

The sirius red dye highlights the collagen fibers in orange red. When examined in polarized light, the thicker collagen fibers appear in reddish tones and the thinner ones appear in greenish tones.

### Statistical analysis

The data obtained in the birefringence analysis in the different groups and experimental periods were analyzed regarding their distribution, being non-parametric. Thus, the Kruskal-Wallis’ tests were applied, followed by Dunn’s test. When analyses between only two groups were necessary, the Mann-Whitney’s test was used.

All statistical tests were evaluated at a significance level of 5% (p < 0.05) and were applied using the GraphPad Prism 5 software (GraphPad, San Diego, CA, United States of America).

## Results

### Macroscopic analysis

In the research, no complications (allergy, fracture or infection) were observed during the postoperative period in any animal.

### Birefringence analysis

To analyze the maturation dynamics of collagen fibers, different birefringent collagen fibers (green, red and yellow) were quantified over periods of 14 and 28 days, under direct light. Regarding the maturation and color of the fibers, it is understood that the red fibers (type I collagen) are more mature fibers, while the green fibers (type III collagen) are immature fibers, that is, new fibers, formed after the creation of defects; yellow fibers are also compatible with type I fibers.

**Figure 3:**
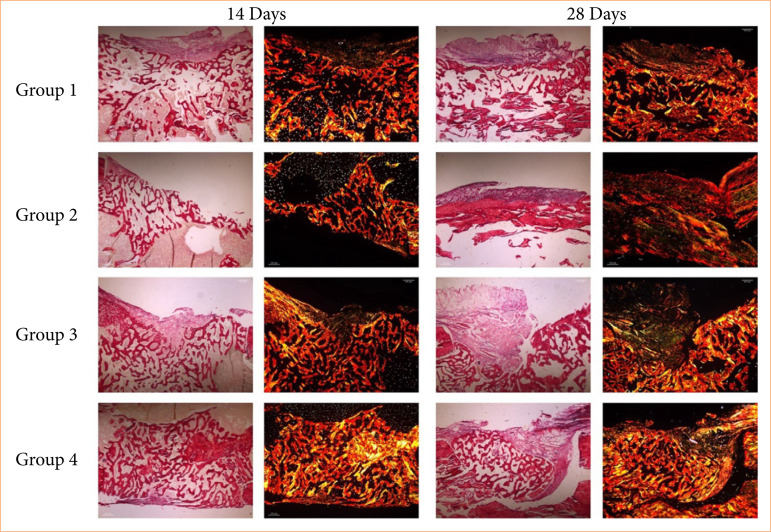
Birefringence analysis of collagen fibers in the experimental periods of 14 and 28 days after the creation of bone defects in groups 1, 2, 3, and 4: representative photomicrographs of the bone tissue of rats from the studied groups, captured under direct and polarized light in which there is a deposition of collagen fibers with visible birefringence in green, yellow and red tones. Picrosirius-red staining; 5x objective, 200 μm-scale bar [553 × 394 mm (300 × 300 DPI)].

Source: elaborated by the authors.

Quantitatively, around 90% of the total area occupied by birefringent fibers was within the red color spectrum, this area being statistically larger in relation to the area occupied by collagen fibers within the green and yellow spectrum, in the four groups studied (Fig. 3).

**Figure 4:**
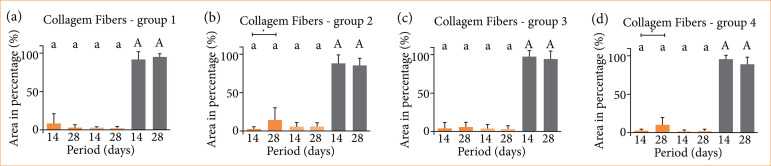
Graphical representation of the means and standard deviation of birefringence data in (%) in different periods (days). Kruskal-Wallis’ and Dunn’s tests (p < 0.05). The red spectrum birefringent fibers were superior to the green and yellow spectrum fibers (aA), in **(a)** group 1, **(b)** group 2, **(c)** group 3 and **(d)** group 4. In the intragroup evaluation, groups **(b)** 2 and **(d)** 4 showed higher values of green birefringent fibers at 28 days when compared to 14 days. *202 × 179 mm (300 × 300 DPI).

Source: Elaborated by the authors.

In the intragroup evaluation, comparing the periods within the same group, the quantification of green birefringent fibers (thin and immature fibers) was shown to be higher in the 28 days when compared to the 14 days, in groups 2 and 4, with values of *p* = 0.0056 and *p* = 0.0091, respectively (Fig. 4).

**Figure 5:**
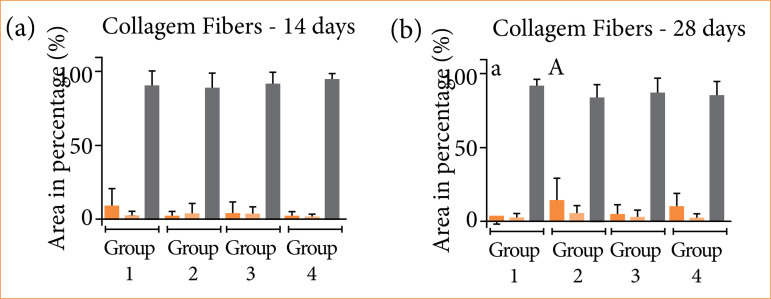
Graphical representation of the means and standard deviation of the birefringence data in (%) in the different periods (days). Kruskal-Wallis’ and Dunn’s tests (p < 0.05). **(a)** 14 days: there was no statistically significant difference. **(b)** 28 days. Group 2 was superior in quantifying green birefringent fibers when compared to group 1. 240 × 92 mm (3,000 × 300 DPI)

Source: Elaborated by the authors.

In the evaluation between the studied groups (intergroup), 14 days after the surgical procedure, there was no statistically significant difference (Fig. 5a). In the 28-day period, group 2 (14.02 ± 15.9%) was superior in quantifying green birefringent fibers when compared to group 1 (3.06 ± 3.24%), with *p* = 0.009 (Fig. 5b). The other groups, throughout the periods, showed a similar pattern, both in terms of the total amount of birefringent fibers.

## Discussion

The main objective of this research was to evaluate the bone repair process in critical bone defects in rats treated with ZA applied systemically associated with local treatment with LLLT. The results found in this research showed that there was a greater quantity of red spectrum birefringent fibers, around 90% of the total area occupied, in the four groups studied. Thus, it was observed that the collagenous matrix that makes up the connective tissue close to the area of the bone defect created in all groups presented a similar pattern in collagen quality, containing an increase in total collagen, with thick fibers and organic matrix maturation, suggesting a low turnover of collagen fibers, even over a period of 14 days.

Furthermore, when comparing the periods, the groups with infrared laser therapy had a greater quantity of fibers with a green spectrum over the 28-day period. It should be noticed that birefringent fibers within the green color spectrum are related to a less organized and more immature matrix, compatible with the initial repair periods.

The bone defect is considered critical when it is not completely filled with bone tissue; in rat tibias, defects equal to or greater than 2 mm in diameter are considered critical^3^^,^ 14. Research carried out to evaluate the bone repair process in critical bone defects submitted to laser therapy associated with ZA is scarce in the literature^3^^,^ 10 ^,^ 11.

The tibia was used as the surgical area due to the ease of replicability and standardization of the experimental model in future studies, as well as because it is a more accessible surgical area when compared to the cranial bones of rats. The medial portion of the tibia was selected as the surgical area, as this area is located far from the growth center, and with less influence from mechanical factors and blood support obstructions, allowing better evaluation of the action of the medication in the bone repair process.

In this study, we did not choose to simulate osteoporosis, as the drug ZA has other indications, including bone metastases. Additionally, ZA has a high action potential that leads to reduced levels of bone turnover after application, when it comes to bone resorption. Therefore, this medication, while maintaining bone density, reduces reabsorption^5^.

There is no exact protocol in the literature regarding the periods and form of application of this medication; in the present study, intravenous injections were applied, alternated weekly. Thus, at the same time that the acid keeps bone mass stable, controlling pathologies related to bone mass, it also interferes with the bone repair process after fractures or critical defects, precisely because it makes bone reabsorption, which is fundamental in the repair process, impossible^3^^,^ 7 ^,^ 12.

Infrared laser therapy was the local treatment option, as the low-level laser stimulates cell proliferation and differentiation, consequently enhancing the repair process. Its effect is related to wavelength, dose, density, duration of irradiation, and frequency^3^^,^ 9 ^,^ 15 ^–^ 18. In this study, the ArAlGa laser with a wavelength of 808 nm was used after the surgical procedure, corroborating previous research^3^^,^ 11 ^,^ 17.

The combination of therapies, bisphosphonates and laser, specifically for the treatment of bone defects, were used in two studies^3^^,^ 17. Garcia et al.^17^ analyzed the repair process in the cap of ovariectomized rats submitted to the use of oral alendronate, associated or not with laser therapy carried out in a single session in the immediate postoperative period. The authors concluded that laser is effective in accelerating repair whether associated with bisphosphonates or not. In contrast, Buchignani et al.^3^ performed the analysis on the tibia of rats, submitted to the application of ZA associated with a low-level laser, through histological analysis, and concluded that the treatment was satisfactory for critical bone defects, stimulating bone formation.

Our research followed a similar methodology. Buchignani et al.^3^, however, used the histochemistry analysis. The results showed that groups 2 and 4 (subjected to laser therapy) presented a greater amount of immature and less organized matrix, over a period of 28 days. This fact occurs because the laser interferes with bone metabolism, as the presence of new collagen fibers was observed, and consequently there is greater bone remodeling, in the late period, in this group^15^^–^ 17.

## Conclusion

We conclude that infrared laser therapy associated with ZA has positive results in stimulating new bone formation in surgically created defects in rats.

## Data Availability

All datasets were generated or analyzed in the current study.
